# Multi-Year Monitoring of the Toxicological Risk of Heavy Metals Related to Fish Consumption by the Population of the Kendari Region (Southeast Sulawesi, Indonesia)

**DOI:** 10.3390/toxics11070592

**Published:** 2023-07-07

**Authors:** Mimie Saputri, Yusnaini Yusnaini, La Sara, Ita Widowati, Thierry Guyot, Denis Fichet, Gilles Radenac

**Affiliations:** 1 UMRi LIENSs 7266 CNRS, La Rochelle Université, 17000 La Rochelle, France; thierry.guyot@univ-lr.fr (T.G.); denis.fichet@univ-lr.fr (D.F.); gilles.radenac@univ-lr.fr (G.R.); 2 Faculty of Fisheries and Marine Sciences, Universitas Diponegoro, Semarang 50275, Indonesia; itawidowati@lecturer.undip.ac.id; 3 Faculty of Teacher Training and Education, Universitas Syiah Kuala, Banda Aceh 23111, Indonesia; 4 Faculty of Fisheries and Marine Sciences, Universitas Halu Oleo, Kendari 93232, Indonesia; yusnaini@uho.ac.id (Y.Y.); la.sara@uho.ac.id (L.S.)

**Keywords:** monitoring, heavy metals, fish, hazard quotient, hazard index, Sulawesi

## Abstract

This study measured the concentrations of Hg, As, Ni, Cd, and Pb in six fish species commonly consumed in Kendari. Samples were bought within local markets from 2012 to 2017 at the end of the dry season. Results showed that mercury concentrations fluctuated between years and within species, except in the *Caranx sexfasciatus*, which showed no significant differences (Kruskall–Wallis, *p*-value > 0.05, df = 5) and an average concentration of 0.371 ± 0.162 µg g^−1^ DW. Arsenic was found in high concentrations across species and years and varied widely in *C*. *sexfasciatus*, the lowest value being 0.32 ± 0.01 µg g^−1^ DW in 2012 and the highest was 5.63 ± 1.89 µg g^−1^ DW in 2017. The highest nickel concentrations were found in 2016 across four of the six species. The fish samples displayed very low cadmium and lead concentrations throughout the study. In addition, the potential human health risk due to fish consumption was assessed. This showed that mercury is the only one of the five metals present in concentrations high enough to individually pose a potential hazard, the only metal likely to be accumulated beyond a safe concentration in Kendari. *Chanos chanos* never posed a toxicological risk based on the results of this research.

## 1. Introduction

### 1.1. Background

Heavy metal contamination poses significant health risks to people worldwide because of their bioaccumulation up to toxic concentrations in living organisms, their impact on cellular functions even at low doses, and their persistence over time [1,2]. Their physicochemical characteristics enable metals to be absorbed, accumulated and transformed by biological functions in living organisms [3,4,5]. Although some metals are essential for metabolic processes, such as copper and iron, others have no known role in humans and can become toxic even at low concentrations, e.g., arsenic, cadmium, mercury or lead. Arsenic-contaminated groundwater in Bangladesh, for example, has affected millions of people over the last two decades [6,7,8].

Coastal communities are particularly at risk of environmental contaminants and heavy metal pollution that impact the health of both aquatic environments and human populations [9,10]. Anthropogenic activities such as urbanization, dredging for coastal development, the deforestation of mangroves and mining activities have increased the number of contaminants present in the marine environment. These contaminants and naturally occurring metals become bio-available for accumulation by marine organisms, which live in continuous contact with pollutants in the water [11,12,13]. These marine organisms, in turn, make the accumulated contaminants available for human absorption when caught and eaten [1,2]. Anthropogenic activities therefore have cascading effects that impact all trophic levels and lead to significant health risks, especially to coastal populations, through the consumption of contaminated seafood products [14,15]. Living organisms can absorb environmental elements such as metals from the air, water, sediments and the food they eat, whether they are beneficial or harmful. Although fish and seafood offer many health benefits, the pollutants present in this category of food can also seriously endanger the health of consumers [16]. For example, fish living near sediments and feeding on detrital organic matter and benthic invertebrates accumulate heavy metals [17]. 

The coastal communities of Indonesia are large fish consumers and local demand for fish is growing [18]. Indonesia is severely affected by anthropogenic pollution and environmental degradation, setting the population at high risk of heavy metal poisoning [19]. Fish offers many benefits to humans as a source of low-fat protein, minerals, vitamins and unsaturated fatty acids essential for cellular functions and organs [2,20]. Fish are also abundantly available in Indonesia and form a major part of the local diet. Yet, as marine organisms are in constant contact with pollutants, fish are also potential vehicles of heavy metal toxicity and a threat to human health. Average fish consumption in Indonesia has increased by more than 30% within a span of four years, from 12.78 kg pers^−1^ year^−1^ in 2011 to over 16.66 kg pers^−1^ year^−1^ in 2015 [18]. This increase may seem moderate but is buffered by the relatively low fish consumption by the vast population of the central Java region. In most parts outside Java, fish consumption is much higher, including the province of Southeast Sulawesi, the region of our study. 

Regarding surface area, Indonesia is the largest archipelagic state [21,22]. Indonesia is also the fourth most populated country in the world with a population spreading over 17,500 islands, of which 6000 islands were recorded to be inhabited by over 264 million people in 2017 [23]. The nation has experienced substantial demographic and economic growth over recent decades [24]. The population increased by 2.79 between 1950 and 2017 [23]. The Indonesian government therefore needs to address the problems of meeting the nutritional needs of the growing population [18]. The GDP per capita has risen from USD 617 in 1985 to over USD 3570 in 2016 [24]. Fishing represented almost 21% of the Indonesian agricultural economy in 2012, almost 3% of the country’s GDP [22]. The country produced nearly 9 million tons of fish, 95% from artisanal activity [25]. In 2012, the Indonesian fishing industry supplied two-thirds of these fish products, while about one-third came from aquaculture [26]. Fishery products (cephalopod fish, crustaceans, etc.) accounted for 54% of the Indonesian population [25]. Indonesia’s strong economic growth may contribute to the growing demand for fish through an increase in the buying power of the local population. 

**Figure 1:**
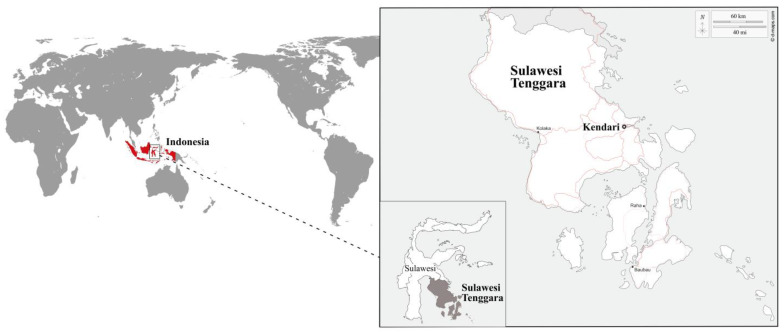
Location of Kendari city, Province of Sulawesi Tenggara, Indonesia.

Our study was conducted in the Kendari agglomeration, which lies at the head of a bay in Southeast Sulawesi, Indonesia (Figure 1). The people of Sulawesi are Indonesia’s second largest fish consumers, consuming an average of 24.9 kg pers^−1^ year^−1^ of marine fish [18]. This area has also displayed remarkable economic growth through palm grove development [27,28], resource exploitation mining [29] and fisheries [25]. The local population is at risk of exposure to heavy metals due to their increasing fish consumption, a risk amplified by the growing economy and demography [30,31].

### 1.2. Mechanisms, Sources and Effects of Heavy Metal Contamination

The concentration of heavy metals accumulated by fish depends on the trophic level of the species [5,32,33] and the conditions of its habitat [12]. Fish, as the most important component of the food chain in aquatic environments, may absorb Heavy metals (HMs) from water and sediment and other species through respiration, feeding and body surface penetration [34]. Pollution by heavy metals and therefore their availability to organisms may vary over the years and seasons [35]. Among aquatic fauna, highly motile fish are not considered good indicators of metallic pollution in marine ecosystems because fish muscle tissue does not readily accumulate contaminants [36,37]. However, muscle tissue effectively indicates a potential toxicological risk to human health because of its substantial presence in most global diets [15,38]. Scientists increasingly present seafood products as a significant source of heavy metal contamination for humans [15,39,40]. In the face of these toxicological risks, global health organizations have established limits on heavy metal concentrations ingested before deleterious effects are developed in humans [23,41]. The dose of heavy metals absorbed depends on the concentration of the element in the source and the modalities of exposure [42]. The duration of exposure and the chemical form of the trace elements are also essential to consider [23,41,43]. It is therefore necessary to monitor the actions of heavy metals, even at low doses, on biological functions and the evolution of concentrations present in commonly consumed fish over time to evaluate potential risks to populations. 

We monitor the five metals most harmful to human health with an oral reference dose (RfD) of less than 0.02 mg kg^−1^ body weight, namely arsenic, cadmium, mercury, nickel and lead [44]. The sources of contamination in coastal environments and the effects of each of these metals are outlined below.

#### 1.2.1. Mercury (Hg)

Mercury pollution in the coastal environment is caused mainly by anthropogenic activities, predominantly mining [45,46]. Its toxicity varies according to its chemical form [13] and methyl mercury is one of the most toxic forms for humans and is introduced by fish consumption [43]. Organisms methylate mercury and accumulate along the food web, particularly in predators such as fish [10]. Several factors, such as size, composition of food in the fish gut, and habitat, had the greatest influence on the mercury levels found in the fish [47]. TRefs. [48,49] suggest that 95% or more of the mercury present in fish muscle is in the form of methyl mercury. A total mercury study therefore provides a conservative approach and assumes—as we have in this paper—that all of the mercury consumed is converted by the species to methylmercury [10]. Mercury is very persistent in the body and it is mainly eliminated via the bile duct. Mercury poisoning causes harmful effects on the liver [50], kidney [51] and brain [52]. Severe neurological symptoms and increased levels of mercury are possibly caused by exposure to inorganic mercury in air, and the consumption of mercury-contaminated fish [53]. The oral reference dose of mercury is 0.0005 mg per kg body weight [41]. 

#### 1.2.2. Arsenic (As)

Arsenic is a by-product of metal mining [54] but is also one of the earth’s crust’s most common naturally occurring elements [43,55]. The routes of infection for humans are principally water and diet [56]. Arsenic has no known function in human physiological activities and causes fetal malformations and infant mortality [43] (WHO—arsenic). The level of toxicity depends on the form of this element. Chronic exposure to inorganic arsenic has carcinogenic effects, and exposure to the organic form deleteriously impacts organs (WHO—arsenic). Acute exposure can lead to gastrointestinal disorders, muscle pain and, in severe cases, death. It is reported by the authors of [57] that only 3% of the total arsenic measured in fish is storable and potentially toxic to humans. The oral reference dose of arsenic is 0.003 mg per kg body weight [41]. 

#### 1.2.3. Nickel (Ni)

There are multiple sources of nickel contamination in the coastal environment, both anthropogenic and natural. According to the Priority List of Hazardous Substances established by the Agency for Toxic Substances and Disease Registry [58], the order of heavy metals in descending order of threat to human health is: As > Pb > Cd > Ni > Zn > Cr > Cu > Mn [59]. Anthropogenic sources include pyrometallurgy and the burning of fossil fuels [60]. Nickel is also released through volcanic ash [61], natural soil erosion and runoff from streams to the sea [62]. Nickel is involved in in lipid metabolism and intensifying hormonal activities [63]. However, nickel inhibits DNA repair and promotes cancer under certain conditions of solubility and bond affinities [43]. The oral reference dose of nickel is 0.02 mg per kg body weight.

#### 1.2.4. Cadmium (Cd)

Cadmium has no known physiological role in humans. It does not exist alone in nature and is frequently bonded with zinc or lead sulfides. Cadmium binds with hemoglobin in erythrocytes and is transported to the liver and the renal cortex. The half-life of cadmium is ±30 years. Detoxification is only possible after completely removing long-term contamination sources [43]. Cadmium poisoning can cause acute renal or pulmonary function disruption and can be lethal. Since cadmium affects mainly the kidneys, the exclusive consumption of muscle tissue limits the exposure and risk of poisoning [64]. The oral reference dose of cadmium is 0.001 mg per kg body weight [41]. 

#### 1.2.5. Lead (Pb)

There are no known metabolic functions of lead in the human body. The digestive system easily absorbs lead: the active transport of lead increases intestinal absorption, amplified by calcium and iron deficiencies because of intense competition with their mucosal receptors [43]. The brain is worst affected because calcium is essential in many neurological processes. In the brain, lead can interfere with mitochondrial function in neurons, preventing cells from functioning correctly. It can also affect the release of neurotransmitters, the way neurons communicate with each other, and change the structure of blood vessels in the brain. Together, these defects can lead to a decreased IQ, learning disabilities, decreased growth, hyperactivity, poor impulse control, and even hearing loss. This is why lead exposure in children is deeply concerning [65]. Pb can cause blood and brain diseases and stomach–intestinal colitis [66,67,68]. In addition, lead reduces the lifespan of red blood cells by altering their membranes [43]. The oral reference dose of lead is 0.004 mg per kg body weight [41]. 

### 1.3. Objective

This study aimed to determine the levels of metallic contamination in fish commonly consumed in the Kendari region of Indonesia and to characterize the toxicological risk of the five metals that pose the most significant toxic risk to humans at low concentrations. We assessed the toxicological risks using internationally recognized indicators: the target hazard quotient (USEPA hazard quotient) and the hazard index (USEPA hazard index). As the first multi-year study of this kind, these quantities will serve as reference levels for the area and can be used to propose recommendations for local health authorities.

## 2. Materials and Methods

### 2.1. Study Species

Six fish species commonly eaten by locals in Kendari were included in this study: *Caranx sexfasciatus* (kuwe as local name), *Chanos chanos* (bandeng), *Epinephelus hexagonatus* (kerapu), *Lethrinus ornatus* (lencam), *Lutjanus gibbus* (kakap) and *Variola albimarginata* (sunu merah). Information on their feeding habits, habitat, trophic level and range was obtained from the Global Fish Information System (Fish Base System) and is summarized in Table 1. The trophic level of each species was estimated from data on their diet.

According to [32], the trophic level of a fish species is strictly herbivorous (level 2.0–2.1) and carnivorous with a preference for fishes and crustaceans (between 4.0 and 4.5). Thus, *V. albimarginata*, *C*. *sexfasciatus* and *E. hexagonatus* are carnivorous species; *L. ornatus* and *L. gibbus* are omnivorous species with a carnivorous preference, consuming a wide variety of prey; and *C. chanos* is an omnivore with an herbivorous preference [33]. These species are distributed broadly throughout the Indo-Pacific, allowing the comparison of our results with other studies in this region.

### 2.2. Sample Selection and Treatment 

Fish were purchased at the Kendari central market to obtain samples that represent those eaten by the locals. Samples were bought every year from 2012 to 2017 at around mid-October, the end of the dry season. The analyses of the samples were carried out annually but following exactly the same protocol from sampling to analysis with the same equipment. Three individuals of similar and marketable size were purchased per species and treated separately (Table 1). We selected fish according to size rather than age to mimic the buying habits of locals and therefore gain a more accurate estimation of risk to the population. A muscle sample was taken from the dorsal part of each fish fillet, a part of the fish that is eaten entirely. The muscle samples were dried in an oven at 45 °C for at least 48 h and ground into a fine homogeneous powder. We used manual grinding with a pestle and mortar for fine homogeneous powder. The powdered samples were stored in inert jars to avoid contamination and placed in a desiccator until analysis to prevent any rehydration of the samples. 

### 2.3. Determining Heavy Metal Concentrations 

Mercury concentrations in the powdered muscle samples (between 5 and 20 mg dry weight, DW) were determined by 254 nm atomic absorption spectrophotometry (Altech AMA-254 made in Czech Republic). Analyses of As, Ni, Cd, Pb were performed with a Varian Vista-Pro ICP-OES made in Australia and a Thermofisher Scientific XSeries 2 ICP-MS made in USA; to this end, aliquots weighing between 60 and 200 mg were digested using a 6:2 (*v*/*v*) 67–70% HNO_3_/34–37% HCl mixture (Fisher, trace metal quality). Acidic digestion of the samples was carried out overnight at room temperature and then in a Milestone microwave oven (30 min with constantly increasing temperature up to 120 °C, then 15 min at this temperature). Each sample was made with up to 50 mL of ultrapure quality water. For samples with a weight of <100 mg, the mixture used was 3:1 (*v*/*v*) 67–70% HNO_3_/34–37% HCl, and the samples were made with up to 25 mL of ultrapure water. Two certified reference materials (CRMs) and one blank, treated and analyzed in the same way as the samples, were included in each analytical batch [69]. A standard certified value sample, TORT-3 (lobster hepatopancreas, NRC, National Research Council Canada), was used to validate the analytical method. Average recovery percentages over the six years of study (2012 to 2017) were compared with the certified values for the five metals. These mean percentages of recovery ranged between 88.7 ± 14.5 for Pb and 105.0 ± 9.4 for As. These values validate the analyses protocol followed. We calculated the averages and standard deviations of the concentrations, expressed in μg g^−1^ DW, and verified their normality using the Shapiro–Wilk test. The data were not normally distributed (*p*-values < 0.05); therefore, nonparametric tests were applied to determine significant differences between samples (Kruskall–Wallis test and Tukey HSD). 

### 2.4. Calculating the Target Hazard Quotients (THQs)

The target hazard quotient (THQ) is a risk index representing the ratio of the daily intake amount to the maximum ingestible amount of metal (the oral reference dose (RfD)) determined by the World Health Organization [70]. This method was available in the USEPA Region III-based concentration table. We evaluated the THQs according to the method used by [71]. The [18] reports that people in Sulawesi consume an average of 23.6 kg of fresh fish per person per year (64.66 g per day). We estimated that they eat fish at least once a day (direct observation of eating habits). The average life expectancy in Indonesia is 73 years for women and 69 for men [72]. The average weight is 52.5 kg for women and 59.1 kg for men. Based on these assumptions, THQ is defined as follows:

THQ = (EF × ED × FIR × C)/(RfD × BW × AT) × 10^−3^ (1)

where EF = exposure frequency (365 days/year); ED = exposure duration over a lifetime (average life expectancy, 73 years for women and 69 for men); FIR = fish intake rate (64.66 g per person per day); C = concentration of metal in the sample (mg kg^−1^ fresh weight); RfD = oral reference dose (mg kg^−1^ day^−1^); BW = average body weight (52.5 kg for women and 59.1 kg for men) and AT = total exposure time over lifetime (EF × ED). A THQ value greater than one indicates that the metal poses a potential risk of adverse health effects in the population (USEPA hazard quotient) [40]. 

### 2.5. Calculating Hazard Indexes (HIs)

The hazard index (HI) is an integrative index that accounts for the simultaneous exposure to different elements in food [73]. Metals can have synergistic effects within the body [40]. To account for these synergistic effects, we evaluated the cumulative health risk by adding the THQs of the metals. The HI in this study is defined as follows:

HI = THQAs + THQCd + THQHg + THQNi + THQPb(2)

An HI value greater than or equal to one indicates a potential for deleterious effects on human health [74,75] (USEPA hazard index).

## 3. Results

### 3.1. Metal Concentrations

Mean metal concentrations per species are reported in Table 2. Mercury concentrations fluctuated between years and within species except in the *Caranx sexfasciatus*, which shows no significant differences (Kruskall–Wallis, *p*-value > 0.05, DL = 5) and an average concentration of 0.371 ± 0.162 μg g^−1^ DW over the study period. The mean mercury concentration in *Chanos chanos* peaked in 2012 at 0.139 ± 0.033 μg g^−1^ DW, is low, not significantly different from 2014 to 2016 (0.053 ± 0.008 μg g^−1^ DW), and was slightly higher in 2017 (0.095 ± 0.009 μg g^−1^ DW). *Epinephelus hexagonatus* had the highest concentrations in 2014 and 2015 with 0.547 ± 0.292 μg g^−1^ DW and 0.620 ± 0.178 μg g^−1^ DW, respectively. The two highest mean mercury concentrations among all species were found in *Lethrinus ornatus* in 2014 (0.872 ± 0.069 μg g^−1^ DW) and *Lutjanus gibbus* in 2015 (0.829 ± 0.278 μg g^−1^ DW). These two species had relatively high intermediate values in 2012, 2013 and 2016 with an average concentration of 0.572 ± 0.054 μg g^−1^ DW. Mercury concentration in *Variola albimarginata* was the greatest in 2013 (0.477 ± 0.280 μg g^−1^ DW).

Arsenic was found in the highest concentrations of all six metals across species and years. Concentrations varied widely in *C. sexfasciatus*, the lowest value being 0.32 ± 0.01 μg g^−1^ DW in 2012 and the highest was 5.63 ± 1.89 μg g^−1^ DW in 2017. Concentrations were also high in 2013 (3.49 ± 0.24 μg g^−1^ DW) and in 2015 (4.09 ± 0.58 μg g^−1^ DW). *C. chanos* and *E. hexagonatus* had very high concentrations in 2013 with 16.20 ± 7.50 μg g^−1^ DW and 14.91 ± 8.65 μg g^−1^ DW, respectively. *L. ornatus* and *L. gibbus* had the highest arsenic concentrations among the six species: 19.47 ± 6.41 μg g^−1^ DW and 39.10 ± 21.39 μg g^−1^ DW, respectively, both in 2014. In *V. albimarginata*, concentrations were comparable over the entire period, averaging 7.96 ± 5.15 μg g^−1^ DW. 

The highest concentrations of nickel were found in 2016 across four of the six species: *C. chanos* (4.33 ± 1.76 μg g^−1^ DW), *E. hexagonatus* (2.56 ± 1.42 μg g^−1^ DW), *L. ornatus* (0.97 ± 0.15 μg g^−1^ DW) and *V. albimarginata* (1.69 ± 1.09 μg g^−1^ DW). Nickel concentrations in *C. sexfasciatus* and *L. gibbus* were not significantly higher than in other years in 2016 because of high variability around relatively high means (6.00 ± 6.25 μg g^−1^ DW and 2.81 ± 3.14 μg g^−1^ DW, respectively). 

The fish samples contained very low concentrations of cadmium and lead throughout the study period, and some concentrations might have been below the detection limit. There is no significant difference between the mean concentrations across the years in any species, except for cadmium concentrations in 2012, which was significantly different in all six species.

### 3.2. Targeted Risk Quotients (THQs)

**Figure 2:**
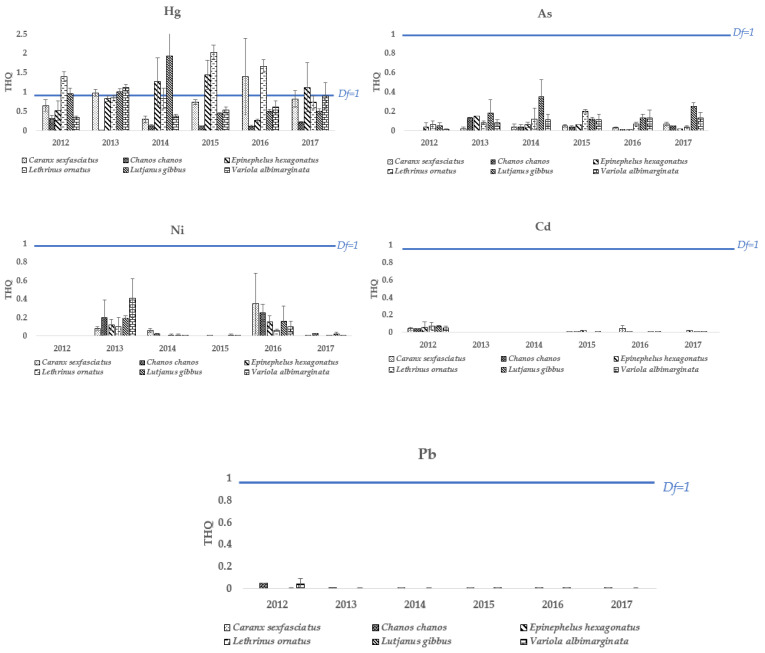
THQ mean value (±SD, n = 3) for years, metals and species.

Reports of the average targeted risk quotients (THQs) of metals according to species were obtained. We combined the average THQs of men and women since we did not find significant differences (Kuskall–Wallis, *p*-value = 0.12, df = 1) (see Figure 2).

Mercury is the only metal which has THQ values exceeding the threshold of 1, which occurs at least twice in all species except *C. chanos*. This species has the lowest THQ for mercury across the years. The maximum THQ for mercury in *C. chanos* was 0.32 ± 0.07 (2012) and the minimum was 0.02 ± 0.00 (2013). The THQs for mercury in *V. albimarginata* do not show a pattern of evolution over the years; the two years in which the THQ was significant were four years apart (2013: 1.11 ± 0.09 and 2017: 0.90 ± 0.34). However, a chronological succession of THQ Hg within species was observed in some cases. Over the first period, from 2012 to 2014, risks increased successively in *L. gibbus* (0.95 ± 0.15, 1.01 ± 0.08, 1.93 ± 0.59). Then, from 2014 to 2016, *L. ornatus* (0.83 ± 0.27, 2.03 ± 0.19, 1.67 ± 0.17) presented the highest risk. Over the last period, the greatest THQs were represented in *C. sexfasciatus* (2016 with 1.40 ± 0.99 and 2017 with 1.40 ± 0.99) and *E. hexagonatus* (2014: 1.27 ± 0.61; 2015: 1.44 ± 0.38; 2017: 1.12 ± 0.64). 

The THQs of the other metals were well below the threshold of 1. The THQs of arsenic are well below the recommended limit probably because the calculation considered only 3% of the total concentration, i.e., the fraction of bioavailability to organisms [57]. The lowest THQs values for arsenic were measured in *C. sexfasciatus* and *C. chanos* in 2012 (<0.01 μg g^1^ DW), while the highest value was in *L. gibbus* in 2014 (0.35 ± 0.18). As for the THQ of nickel, 2013 and 2016 are the only years that stand out, with higher values than in other years. The maximum THQs of nickel were in *V. albimarginata* in 2013 (0.41 ± 0.21) and *C. sexfasciatus* in 2016 (0.35 ± 0.33). The THQs of cadmium and lead present no risks across species and years. 

### 3.3. Hazard Indexes (HIs)

The average hazard index (HI) values per year for each species are shown in Table 3. All species had potential risk values greater than 1 for at least one year, except *C. chanos*, which has a very low-risk potential regardless of the year (an overall average of 0.32 ± 0.10). The years 2012, 2014 and 2015 represent low-risk periods because only two species per year presented high risks: in 2012, *L. ornatus* (1.53 ± 0.11) and *L. gibbus* (1.07 ± 0.18); in 2014, *E. hexagonatus* (1.34 ± 0.62) and *L. gibbus* (2.29 ± 0.52); and in 2015, *E. hexagonatus* (1.52 ± 0.38) and *L. ornatus* (2.23 ± 0.22). The year 2017 presents an intermediate risk because half of the fish species presented risks higher than the threshold: *C. sexfasciatus* (0.89 ± 0.21), *E. hexagonatus* (1.17 ± 0.65) and *V. albimarginata* (1.04 ± 0.38). Finally, 2013 and 2016 posed the highest risks; two-thirds of the species had high HIs greater than 1. For both these years, *C. sexfasciatus* (1.08 ± 0.13, 1.82 ± 1.02), *L. gibbus* (1.37 ± 0.25, 0.80 ± 0.20) and *V. albimarginata* (1.59 ± 0.33, 0.85 ± 0.26) had high cumulative health risks, while only *E. hexagonatus* (1.10 ± 0.13) in 2013 and *L. ornatus* (1.80 ± 0.18) in 2016.

**Figure 3:**
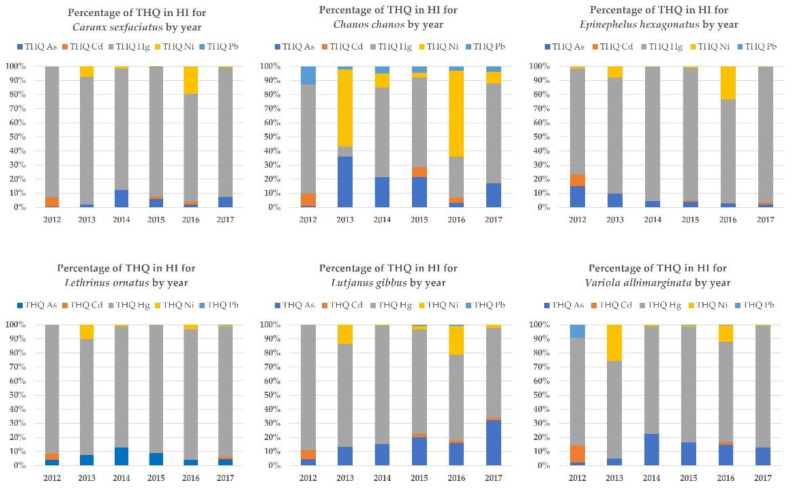
Part of each metal-specific THQ (in percentage) in the global HI for different species and years. Colors for the various metals are given inside the figure.

The contribution of each metal’s THQ to the total potential risk to human health (HI) is shown in Figure 3. Mercury is by far the most significant risk contributor across all years and species except in *C. chanos* in 2013 and 2016 where it is exceeded by nickel. The risk posed by nickel is present in variable percentages across species but in higher proportions in these two years. The THQ of arsenic is also variable across all years and all species but its contribution to HI is the highest in *C. chanos* in 2013 and 2015 (>20%). Arsenic contri- buted increasingly to the HI of *L. gibbus* over the years, exceeding 20% in 2015 and 2017. In *E. hexagonatus*, *L. ornatus* and *V. albimarginata*, however, a relative decrease in the percentage contribution of arsenic is observed from 2014. A similar trend exists in *C. sexfasciatus*, except for a notable increase in 2017. The percentage contributions of cadmium and lead are minor, relative to the other metals. The contribution to the HI by cadmium is significant only in 2012 (±10%) for all species. The contribution to the HI by lead is only noticeable in *C. chanos* (±5%) across all years, and in *V. albimarginata* in 2012 (10%).

### 3.4. Trophic Level and Hazard Indexes (HIs)

**Figure 4:**
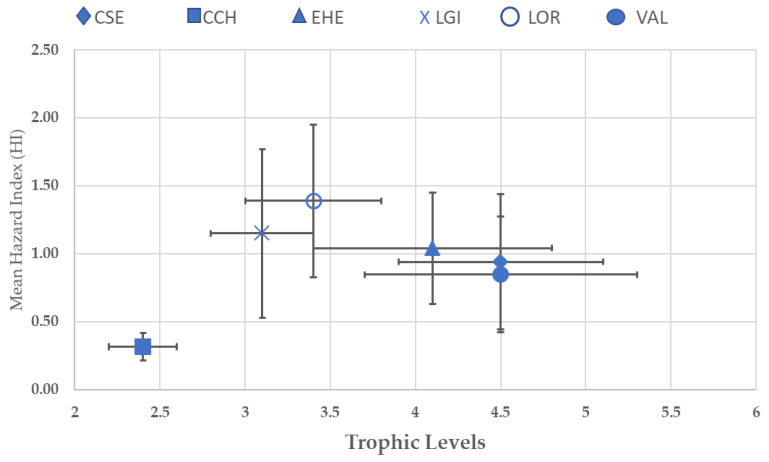
Correlation between the trophic level and the HI for each species.

Figure 4 shows the relationship between the trophic level of each species and the corresponding mean HI values. We found no significant correlation between the mean HI (over the six years) and the trophic level of each species (Spearman, *p*-value = 0.27, DL = 5, r^2^ = 0.007). *C. chanos* has the lowest trophic level (2.4 ± 0.2, Table 3) and the lowest mean HI value (0.31 ± 0.13) of all species. *C. sexfasciatus*, *E. hexagonatus* and *V. albimarginata* form a group in which the mean HI values are intermediate (0.92 ± 0.65, 1.12 ± 0.57 and 0.75 ± 0.67, respectively) but trophic levels are highest (4.5 ± 0.6, 4.1 ± 0.7, 4.5 ± 0.8, respectively; Table 1). *L. ornatus* has the highest average HI value (1.43 ± 0.56) but an intermediate trophic level (3.4 ± 0.4, Table 1). *L gibbus* has an intermediate HI (1.12 ± 0.65) and an intermediate trophic level (3.1 ± 0.3, Table 1).

## 4. Discussion

The mercury (Hg) concentrations measured in our studies are (effectively) lower than those recommended by authorities such as the FAO (2003) or certain reviews [76]. For example, our results on mercury presented a maximum concentration of 2.5 μg g^−1^ DW that is twice weaker than the recommended maximum value (5 μg g^−1^ DW) even though, here, a more integrative approach considering toxicological parameters (RfD; oral refe-rence dose), but also human biology and sociology indicators, have been deliberately chosen. Thus, we believe that these toxicological indices better represent the risk of these foods for the health of these populations. Of all the metals, the Kendari population is most at risk of mercury poisoning. Mercury is the only one of the five metals present in concentrations high enough to pose a potential hazard individually, the only metal likely to accumulate beyond a safe concentration in the average Kendari. This was not the case in similar studies, which reported only concentrations within safe limits [33,77,78]. The THQs of mercury exceed the threshold of one several times in all but one species, *Chanos chanos*, which never exceeds the threshold and poses relatively little risk of mercury toxicity. When considered individually, no other metal poses a toxic risk; the THQs of arsenic, cadmium, nickel and lead are below the recommended limit. The peaks of nickel THQs in years 2013 and 2016 coincide with an increase in mining activities in Indonesia and a change in nickel mining policies in recent years. 

The differences in their diet can partially explain the differences in toxicity between fish species. The size of fish was our reference to slightly homogenize the age of the sampled individuals. Metal concentrations can be confirmed through factors such as the level of environmental contamination and the duration based on [79]. As a result, as the organization grows, larger individuals (i.e., older individuals) are expected to accumulate higher metal concentrations than smaller individuals (i.e., younger individuals). Another factor potentially affecting metal bioaccumulation is the species richness and biomass of the recipient environment [80]. Their diet and trophic level determine the accumulation rate of heavy metals and therefore the biomagnification of these elements throughout the food web [35]. Polychaetes are very good bioaccumulators of metals and can be used in environmental biomonitoring studies [36]. Crustaceans and echinoderms are also good indicators of the bioaccessibility of heavy metals in the environment [81]. Heavy metals can be accumulated by fish from food, water and sediments [82]. The fish are reliable indicators of the levels of heavy metal contamination in aquatic environments [83] because the concentrations of metals in fish often correspond with the levels in the soil and water of a specific aquatic environment from where they are sourced [84] and their duration of exposure [85]. In this study, we did not use data on heavy metal concentrations within water and sediment because the main purpose of this research protocol is to assess the toxic risk samples of fish that were sold in traditional Kendari markets and not to study the correlation between water and sediment parameters and heavy metal content in fish in the Kendari area. Fish have a higher risk of accumulating heavy metals when they prey on good bioaccumulators, like polychaetes, crustaceans and echinoderms. However, we found no significant correlation between the trophic level and the risk of harmful effects on human health (Figure 4). Diet therefore does not identify species at risk, except in the case of *C. chanos*, a low trophic-level herbivore, which undoubtedly poses no risk to human health in the Kendari area throughout the follow-up period—with THQ and HI values remaining well below the risk threshold. Of the six species studied, *C. chanos* is the only one that can be bred in captivity through aquaculture and therefore be reared on a food supply of controlled quality [86].

To understand the potential impact of the consumption of the various fish species by the locals in Kendari, it is necessary to consider their cost to household income. The gross domestic product (GDP) per capita has increased by an annual average of 2.9% between 1999 and 2005 [87], which has led to an increase in fish consumption in Indonesia [18]. However, it remains likely that the cost of buying a species limits its consumption. The fish species with high toxic risks were more expensive and less accessible to many consumers. *Lutjanus gibbus*, for example, presents substantial risks and was sold for IDR 45,000 each in 2022. *C. chanos* has a low potential risk and costs only IDR 25,000 per individual in 2023. Species with a high-risk index were not only the most expensive, but most desired for their taste by the Kendari population. 

Aside from the cost of fish and local preferences, we must consider the possibility of reducing the intake of metal elements in food. Many studies support that cooking is the best way to minimize food-contaminant intake. Cooking methods have been shown to have varying effects on meat quality, leading to differences in nutrient composition and trace element concentration [88]. A study by the authors of [89] evaluated the impact of various cooking methods on total mercury concentration in fish and found significant differences between roasting, boiling and frying. Scavenging and carnivorous fish lose a lot of mercury when cooked. Omnivorous and herbivorous fish experienced a sharp decline in mercury concentration when grilled. Several studies have shown the effects of different types of cooking on the bioavailability of trace elements in two farmed marine species, *L. japonicus* and *P. majo.* Boiling, steaming, frying and broiling reduced the bioavailability of arsenic, cadmium, copper, iron, selenium and zinc [90]. The last two methods are more efficient than the second. Studies on the effects of cooking on *C. gariepinus* supports the findings of [90,91], which report that all cooking methods reduce the availability of heavy metals in the flesh. The preferred cooking method depends on the trace elements to be guarded against and the concentrations present in the species. Omnivorous species pose the most significant risk in our study because of their high mercury concentration; therefore, according to [89], the most effective cooking method to reduce toxic risk is grilling. In this publication, we admit that the cooking method does not influence the metal concentrations in the samples [92]. 

The accumulation of dangerous levels of heavy metals can be averted by cooking fish well and diversifying protein sources [93], taking care to eat fish of lower trophic levels. The species to be favored are the primary consumers (planktivorous and microphages), usually available at lower costs. In this study, *C. chanos* presented negligible risks, regardless of the year. This species is accessible at a low price for the population, due to the species being bred in aquaculture facilities [86]. This reduces the accumulation of heavy metals and contaminants through isolation from marine environments and better food quality control. Predators with varied diets, such as *Lethrinus ornatus*, are to be limited because their diverse prey types and sites increase the risk of heavy metal accumulation [94,95]. Nevertheless, fish are not the only source of heavy metal toxicity; other foods can also contain high concentrations, including rice, which is found in most Asian diets [73,96]. More comprehensive analyses of population diets, beyond the scope of this work, are necessary for the assessment of overall risks associated with the consumption of common foods in high-risk areas.

### Heavy Metal Concentrations in the Context of Other Studies

A comparison of heavy metal concentrations (mg kg^−1^ DW) found in fish from other study areas in the Indo-Pacific region are presented in Table 4.

Total mercury concentrations fluctuate in our study depending on the species studied and the year except for *Caranx sexfasciatus*, which shows no significant difference between 2012 and 2017 (Table 2). This species contains on average 0.37 ± 0.26 μg g^−1^ DW, a similar concentration to that obtained in the same species in Malaysia with 0.29 μg g^−1^ DW [33]. However, the study by the authors of [97] reported a mercury concentration ten times higher for *Caranx ignobilis* with 2.01 ± 0.82 μg g^−1^ DW, also in Malaysia. For *Chanos chanos*, the highest concentration, measured in 2012 (0.139 ± 0.033 μg g^−1^ DW), is ten times higher than that obtained by the authors of [98] in the Philippines, with 0.013 μg g^−1^ DW. Their result was similar to the concentration level we found in *C. chanos* the following year (2013, 0.011 ± 0.001 μg g^−1^ DW). *Epinephelus hexagonatus* had the highest mercury concentrations in 2015 with 0.620 ± 0.178 μg g^−1^ DW. This value is an order of magnitude greater than the concentration measured for *E. sexfasciatus* in the Strait of Malacca (0.015 μg g^−1^ DW; [77]) and two to three times higher than that of *E. quoyanus* on the east coast of India (0.237 ± 0.003 μg g^−1^ DW; [94]). *Lethrinus ornatus* had a high mercury concentration in 2015, 0.872 ± 0.069 μg g^−1^ DW, a value four times higher than that measured by the authors of [94] for *L. lentjan* on the east coast of India (0.212 ± 0.002 μg g^−1^ DW). A lower concentration of 0.48 ± 0.08 μg g^−1^ DW for *L. lentjan* was found by the authors of [103] on the east coast of Malaysia. The species with one of the highest concentrations is *Lutjanus gibbus* with 0.829 ± 0.278 μg g^−1^ DW in 2014. This concentration is double that of the concentration obtained by the authors of [33] in the same species in Malaysia (0.436 μg g^−1^ DW). Similarly, the concentration obtained by the authors of [77] in the Strait of Malacca for *L. argentimeculatus* is more than twenty-three times lower than the concentration in this study (0.007 ± 0.000 μg g^−1^ DW). 

Compared to others references, arsenic was found in the highest concentration in our study, which is not surprising given that arsenic is one of the most common naturally occurring elements in the earth’s crust (see Section 1.2.2; [43,55]). *Lethrinus ornatus* and *Lutjanus gibbus* had the highest concentrations of all species in 2014 with 19.47 ± 6.41 μg g^−1^ DW and 39.10 ± 21.39 μg g^−1^ DW, respectively. These concentrations are similar to those obtained by the authors of [99] in New Caledonia for *L. laticaudis* (16.7 ± 5.75 μg g^−1^ DW) but are five times higher for *L. argentimaculatus* (7.70 ± 0.76 μg g^−1^ DW) in the same study. Similarly, the authors of [77] obtained values nearly 40 times lower than for *L. argentimaculatus* in the Strait of Malacca (1.55 ± 0.07 μg g^−1^ DW). Arsenic concentrations were 2 to 4 times lower than results from an unpublished study of ours, which found 8.54 ± 3.52 μg g^−1^ DW in *L. ornatus* and 10.87 ± 5.13 μg g^−1^ DW in *L. gibbus*.

The average nickel concentration is high in all of the samples from 2016. *C. chanos* in 2014 in Flores, Indonesia has the highest mean concentration with 4.33 ± 1.76 μg g^−1^ DW in 2016, which is nearly ten times higher than that obtained by the authors of [101] on the east coast of Java (0.48 ± 0.09 μg g^−1^ DW) but only half of that measured by [102] on the west coast of Taiwan (9.04 ± 8.08 μg g^−1^ DW). The high concentrations obtained in 2016 can be explained by the adjustments of operating policies where new nickel extraction sites were being set up on the islands of Indonesia, particularly in Sulawesi. It takes over a year to observe the effects of implemented environmental policies, such as waste reduction and the treatment of water used for extraction [29]. Changes in nickel extraction policies were enacted, starting from 2015 and continued until 2019; environmental programs have put forward management plans for the resulting waste [29]. New mining standards were implemented to reduce the ecological impact of extractions, and the number of inspectors implementing waste management requirements was increased [29]. 

The maximum concentration of cadmium was measured in 2012 for *L. ornatus* with 0.06 ± 0.04 μg g^−1^ DW. This is relatively low value but is almost twice that which was obtained in *L. rubrioperculatus* by the authors of [4] on the Hainan coast in China (0.006 ± 0.003 μg g^−1^ DW) and half of the concentration obtained by the authors of [78] in *L. lentjan* on the east coast of India (0.13 ± 0.01 μg g^−1^ DW).

All species have low lead concentrations, many even below the lead detection limit. The lead concentrations of *C. chanos* are much lower than those measured by the authors of [103], whereby their lowest concentration was 1.447 μg g^−1^ in the north of Jakarta. Similarly, lead concentrations obtained by the authors of [104] in *C. chanos* in the Philippines were double that of our findings, with 0.164 ± 0.003 μg g^−1^ DW. 

## 5. Conclusions

According to the results presented here, mercury is the only metal of the five studied currently showing toxicological risks for consumers. The concentration and toxicological index (THQ) values of mercury exceeded the threshold value in most fish species, indicating that the average person living in Kendari is at risk of accumulating enough mercury over their lifetime to cause harmful effects on their health. Fish with higher trophic levels presented more significant toxicological risks than the primary consumer, *Chanos chanos* (Forsskål, 1775). Since no significant and clear relationship was found between the same trophic levels and fish toxicity, we recommend that the consumption of omnivorous and carnivorous fish (mid to high trophic levels) be varied and kept to a minimum. Fish of low trophic levels should be favored. The exploitation of fishery resources and aquaculture development, which supplement the production of fish of lower trophic levels and therefore of lower toxic risks, can help meet the fish demands in the growing population of Kendari and the Sulawesi region. The aquaculture of milkfish (*C. chanos*) has been proposed by the FAO as an option to address food security and meet the increasing food protein demand in the Pacific Island countries and territories (PICTs) [105]. It is one of many species raised to ensure food security and to meet the current demand for dietary protein in the Western Pacific region [106]. Monitoring the abundance and availability of potentially toxic elements in the environment and prominent food sources is essential to understand the risks to both humans and the environment and, therefore, to make informed recommendations and actions to protect the health of both consumers and the environment which they depend on.

## Figures and Tables

**Table 1:** General information about the sampled species, including the habitat of the species, the estimated trophic level and the feeding habits.

Species	Common Name	Habitats	Trophic Level	Feeding Habits
*Caranx sexfasciatus*	Bigeye trevally	Pelagic, oceanic and coastal waters, coral reefs	4.5 ± 0.6	Fishes, crustaceans
*Chanos chanos*	Milkfish	Benthopelagic, coastal waters	2.4 ± 0.2	Plankton
*Epinephelus hexagonatus*	Starspotted grouper	Epibenthic, coastal waters	4.1 ± 0.7	Fishes, crustaceans
*Lethrinus ornatus*	Ornate emperor	Demersal, various habitats (sandy, sea-grass meadows, coral reefs)	3.4 ± 0.4	Fishes, crustaceans, mollusks, annelids
*Lutjanus gibbus*	Humpback red snapper	Benthopelagic in coral reefs	3.1 ± 0.3	Fishes, crustaceans, cephalopods, echinoderms.
*Variola albimarginata*	White-edged lyretail	Benthopelagic in coral reefs	4.5 ± 0.8	Fishes

**Table 2:** Mean metal concentrations (±SD, n = 3, in µg g^−1^ DW) according to species. Bold values are mean values significantly higher from each other’s (Kruskall-Wallis, *p* < 0.05). a, b, c, d: homogeneous groups after a Tukey-test for species and years.

Year	Hg	As	Ni	Cd	Pb
***Caranx sexfasciatus***					
2012	0.279 ± 0.072 ^a^	0.32 ± 0.01 ^a^	ND (0.02)	**0.04 ± 0.02 ^b^**	ND (0.03)
2013	0.421 ± 0.082 ^a^	3.49 ± 0.24 ^bcd^	0.24 ± 0.18 ^a^	ND (0.02)	ND (0.02)
2014	0.126 ± 0.032 ^a^	1.57 ± 0.55 ^ab^	0.09 ± 0.09 ^a^	ND (0.02)	ND (0.02)
2015	0.318 ± 0.002 ^a^	4.09 ± 0.58 ^cd^	0.05 ± 0.03 ^a^	0.01 ± 0.00 ^a^	0.01 ± 0.00 ^a^
2016	0.601 ± 0.476 ^a^	2.76 ± 0.45 ^ac^	6.00 ± 6.25 ^a^	0.03 ± 0.04 ^a^	0.01 ± 0.00 ^a^
2017	0.484 ± 0.306 ^a^	**5.63 ± 1.89 ^d^**	0.11 ± 0.06 ^a^	ND (0.01)	ND (0.01)
***Chanos chanos***					
2012	**0.139 ± 0.033** ^c^	0.33 ± 0.01 ^a^	ND (0.02)	**0.03 ± 0.00** ^b^	0.18 ± 0.14 ^a^
2013	0.011 ± 0.001 ^a^	**16.20 ± 7.50** ^b^	0.81 ± 0.23 ^a^	ND (0.02)	0.05 ± 0.07 ^a^
2014	0.055 ± 0.008 ^ab^	2.82 ± 1.27 ^a^	0.22 ± 0.03 ^a^	ND (0.02)	0.03 ± 0.01 ^a^
2015	0.052 ± 0.003 ^ab^	3.47 ± 0.73 ^a^	0.11 ± 0.07 ^a^	0.01 ± 0.00 ^a^	0.03 ± 0.01 ^a^
2016	0.051 ± 0.014 ^ab^	1.14 ± 0.11 ^a^	**4.33 ± 1.76** ^b^	0.01 ± 0.00 ^a^	0.04 ± 0.01 ^a^
2017	0.095 ± 0.009 ^bc^	4.54 ± 0.28 ^a^	0.45 ± 0.07 ^a^	ND (0.01)	0.04 ± 0.01 ^a^
***Epinephelus hexagonatus***					
2012	0.242 ± 0.095 ^ab^	5.10 ± 5.68 ^a^	ND (0.02)	0.07 ± 0.07 ^a^	ND (0.03)
2013	0.359 ± 0.120 ^ab^	**14.91 ± 8.65 ^b^**	0.39 ± 0.27 ^a^	ND (0.02)	ND (0.02)
2014	0.547 ± 0.292 ^ab^	9.03 ± 3.26 ^ab^	0.10 ± 0.04 ^a^	ND (0.02)	ND (0.02)
2015	**0.620 ± 0.178** ^b^	5.27 ± 0.73 ^a^	0.09 ± 0.07 ^a^	0.01 ± 0.00 ^a^	0.01 ± 0.00 ^a^
2016	0.112 ± 0.018 ^a^	1.27 ± 1.42 ^a^	**2.56 ± 1.42** ^b^	ND (0.01)	ND (0.03)
2017	0.312 ± 0.078 ^ab^	1.88 ± 0.24 ^a^	0.06 ± 0.02 ^a^	0.01 ± 0.00 ^a^	0.01 ± 0.00 ^a^
***Lethrinus ornatus***					
2012	0.601 ± 0.046 ^bc^	5.19 ± 3.47 ^a^	ND (0.02)	**0.06 ± 0.04** ^b^	ND (0.03)
2013	0.360 ± 0.107 ^ab^	7.28 ± 7.99 ^ac^	0.43 ± 0.08 ^a^	ND (0.02)	ND (0.02)
2014	0.358 ± 0.128 ^ab^	**19.47 ± 6.41** ^c^	0.27 ± 0.35 ^a^	ND (0.02)	ND (0.02)
2015	**0.872 ± 0.069** ^d^	16.75 ± 1.49 ^bc^	0.08 ± 0.00 ^a^	ND (0.01)	0.01 ± 0.00 ^a^
2016	0.718 ± 0.065 ^cd^	6.01 ± 2.16 ^ab^	**0.97 ± 0.15** ^b^	ND (0.01)	ND (0.01)
2017	0.351 ± 0.100 ^a^	3.03 ± 0.57 ^a^	0.10 ± 0.03 ^a^	0.01 ± 0.00 ^a^	0.01 ± 0.00 ^a^
***Lutjanus gibbus***					
2012	0410 ± 0.067 ^a^	4.16 ± 2.70 ^a^	ND (0.02)	**0.06 ± 0.01** ^b^	ND (0.03)
2013	0.433 ± 0.135 ^ab^	22.53 ± 10.47 ^ab^	0.50 ± 0.65 ^a^	ND (0.02)	ND (0.02)
2014	**0.829 ± 0.278** ^b^	**39.10 ± 21.39** ^b^	0.09 ± 0.05 ^a^	ND (0.02)	ND (0.02)
2015	0.189 ± 0.008 ^a^	10.27 ± 1.98 ^a^	0.26 ± 0.13 ^a^	0.01 ± 0.00 ^a^	0.01 ± 0.00 ^a^
2016	0.210 ± 0.021 ^a^	10.92 ± 4.14 ^a^	2.81 ± 3.14 ^a^	0.01 ± 0.00 ^a^	0.02 ± 0.00 ^a^
2017	0.386 ± 0.162 ^a^	21.52 ± 3.89 ^ab^	0.30 ± 0.41 ^a^	0.01 ± 0.00 ^a^	ND (0.01)
***Variola albimarginata***					
2012	0.148 ± 0.011 ^a^	0.86 ± 0.71 ^a^	LD (0.02)	**0.05 ± 0.02** ^b^	0.14 ± 0.20 ^a^
2013	**0.477 ± 0.280** ^b^	7.94 ± 7.43 ^a^	1.35 ± 1.00 ^ab^	ND (0.02)	ND (0.02)
2014	0.163 ± 0.018 ^a^	7.47 ± 3.26 ^a^	0.07 ± 0.02 ^a^	ND (0.02)	ND (0.02)
2015	0.229 ± 0.036 ^ab^	9.15 ± 6.01 ^a^	0.09 ± 0.04 ^a^	ND (0.01)	0.01 ± 0.00 ^a^
2016	0.261 ± 0.075 ^ab^	10.93 ± 7.76 ^a^	**1.69 ± 1.09** ^b^	0.01 ± 0.00 ^a^	0.02 ± 0.00 ^a^
2017	0.214 ± 0.030 ^ab^	11.40 ± 5.73 ^a^	0.11 ± 0.04 ^a^	ND (0.01)	ND (0.01)

ND = Not detected.

**Table 3:** HI mean values for species and years. Bold values are higher than reference value of 1 considering the SD (n = 3); a, b, c, d: homogeneous groups after a Tukey test for species and years. CSE = *Caranx sexfasciatus*; CCH *= Chanos chanos*; EHE = *Epinephelus hexagonatus*; LOR = *Lethrinus ornatus*; LGI *= Lutjanus gibbus*; VAL = *Variola albimarginata*.

Years	CSE	CCH	EHE	LOR	LGI	VAL
2012	0.70 ± 0.16 ^a^	0.42 ± 0.11 ^b^	0.70 ± 0.17 ^ab^	**1.53 ± 0.11 ^b^**	**1** **.07 ± 0** **.18 ^b^**	0.45 ± 0.09 ^a^
2013	**1.08 ± 0.13 ^ab^**	0.36 ± 0.30 ^ab^	**1.10 ± 0.13 ^ac^**	**1.02 ± 0.19 ^a^**	**1.37 ± 0.25 ^b^**	**1.59 ± 0.33** ^d^
2014	0.34 ± 0.08 ^a^	0.20 ± 0.04 ^a^	**1.34 ± 0.62 ^bc^**	**0.97 ± 0.26** ^a^	**2.29 ± 0.52 ^c^**	0.50 ± 0.08 ^ab^
2015	0.81 ± 0.07 ^a^	0.19 ± 0.02 ^a^	**1.52 ± 0.38** ^c^	**2.23 ± 0.22 ^c^**	0.59 ± 0.04 ^a^	0.65 ± 0.05 ^ab^
2016	**1.82 ± 1.02 ^b^**	0.41 ± 0.10 ^b^	0.42 ± 0.07 ^a^	**1.80 ± 0.18 ^b^**	**0.80 ± 0.20 ^ab^**	**0.85 ± 0.26 ^bc^**
2017	**0.89 ± 0.21 ^a^**	0.31 ± 0.02 ^ab^	**1.17 ± 0.65 ^ac^**	0.78 ± 0.17 ^a^	0.78 ± 0.08 ^ab^	**1.04 ± 0.38 ^c^**

**Table 4:** Comparison of heavy metal concentrations (mg kg^−1^ DW) found in fish from other study areas in the Indo-Pacific region.

Heavy Metals (HMs)	Species	Study Area	Concentration HMs (mg kg^−1^ DW)	References
Mercury (Hg)	*Caranx sexfasciatus*	Kendari, Indonesia	0.370	This study
		Malaysia	0.290	[33]
	*Caranx ignobilis*	Malaysia	0.210	[97]
	*Chanos chanos*	Kendari, Indonesia	0.139	This study
		Philippines	0.013	[98]
	*Epinephelus hexagonatus*	Kendari, Indonesia	0.620	This study
	*Epinephelus sexfaciatus*	Straits of Malacca	0.015	[77]
	*Epinephelus quoyanus*	India	0.237	[94]
	*Lethrinus ornatus*	Kendari, Indonesia	0.872	This study
	*Lethrinus lentjan*	India	0.212	[94]
		Malaysia	0.048	[99]
	*Lutjanus gibbus*	Kendari, Indonesia	0.829	This study
		Malaysia	0.436	[33]
	*Lutjanus argentimeculatus*	Straits of Malacca	0.007	[77]
Arsenic (As)	*Lethrinus ornatus*	Kendari, Indonesia	19.47	This study
		Flores, Indonesia	8.54	Unpublished study, 2014
	*Lethrinus laticaudis*	New Calidonia	16.70	[100]
	*Lethrinus argentimeculatus*	New Calidonia	7.70	[100]
		Straits of Malacca	1.55	[77]
	*Lutjanus gibbus*	Kendari, Indonesia	39.10	This study
		Flores, Indonesia	10.87	Unpublished study, 2014
		Taiwan	9.04	[101]
Cadmium (Cd)	*Lethrinus ornatus*	Kendari, Indonesia	0.060	This study
	*Lethrinus rubrioperculatus*	Hainan, China	0.006	[4]
	*Lethrinus lentjan*	India	0.130	[94]
Lead (Pb)	*Chanos chanos*	Kendari, Indonesia	0.050	This study
		Jakarta, Indonesia	1.447	[102]
		Philippines	0.164	[103]

## Data Availability

Not applicable.
